# Serum Cystatin C Predicts Stroke Clinical Outcomes at 1 Year Independent of Renal Function

**DOI:** 10.3389/fneur.2021.676872

**Published:** 2021-08-11

**Authors:** Yarong Ding, Liping Liu, Zimo Chen, Hao Li, Yuesong Pan, Junfeng Wang, Xia Meng, Jinxi Lin, Jing Jing, Xuewei Xie, Xianglong Xiang, Yongjun Wang

**Affiliations:** ^1^Department of Neurology, Beijing Tiantan Hospital, Capital Medical University, Beijing, China; ^2^China National Clinical Research Center for Neurological Diseases, Beijing, China; ^3^Center of Stroke, Beijing Institute for Brain Disorders, Beijing, China; ^4^Beijing Key Laboratory of Translational Medicine for Cerebrovascular Disease, Beijing, China; ^5^Julius Center for Health Sciences and Primary Care, University Medical Center Utrecht, Utrecht University, Utrecht, Netherlands

**Keywords:** cystatin C, renal function, biomarker, ischemic stroke, clinical outcomes

## Abstract

**Objective:** Serum cystatin C (CysC) is a sensitive marker of renal function to predict cardiovascular diseases. We aimed to investigate the predictive value of CysC for clinical outcomes independent of renal function in patients with acute ischemic stroke (AIS).

**Methods:** We measured serum CysC levels in 10,256 AIS patients from Third China National Stroke Registry (CNSR-III). The primary outcome was a combination of all-cause mortality and major disability (modified Rankin scale score, 3–6). Secondary outcomes included stroke recurrence and combined vascular events at 1 year. Outcomes were analyzed using logistic regression and Cox proportional hazards models, respectively.

**Results:** The median CysC of included patients was 0.95 mg/l (interquartile range, 0.83–1.10 mg/l). A U-shaped association was observed between CysC and primary outcome (all-cause mortality or major disability) [quartile (Q)1 vs. Q2: adjusted odds ratio (aOR) 1.29, 95% CI 1.06–1.58, *p* = 0.012; Q3 vs. Q2: aOR 1.12, 95% CI 0.93–1.35, *p* = 0.242; Q4 vs. Q2: aOR 1.35, 95% CI 1.10–1.65, *p* = 0.004]. A similar trend also existed in “preserved renal function” patients. Adding CysC to a model containing conventional risk factors improved the model performance with integrated discrimination improvement (IDI) of 0.13% (*p* = 0.016) and net reclassification index (NRI) of 13.10% (*p* <0.001) for primary outcome. No significant association was observed for stroke recurrence or combined vascular event rate in different CysC quartiles.

**Conclusions:** CysC showed a U-shaped correlation with 1-year stroke clinical outcome, suggesting that serum CysC may not only be a simple candidate marker of renal function.

## Introduction

Cystatin C (CysC), a protein inhibitor of cysteine protease, was generally considered an alternative to creatinine for kidney function measurement (1). It was also reported as a predictive marker of cardiovascular diseases (CVDs) (2, 3). Besides, CysC was independently associated with cerebral artery stenosis and mortality in stroke or CVD patients with estimated glomerular filtration rate (eGFR) ≥60 ml/min/1.73 m^2^ (4, 5). Thus, it is suggested that CysC may act in versatile roles rather than a single index for glomerular filtration.

Since serum CysC was considered a marker of endothelial dysfunction in the glomerulus and throughout the vascular tree, elevated CysC levels may indicate a higher degree of cerebral vessel damage (6). On the other hand, since CysC is a potent competitive inhibitor of cysteine proteases, low levels of CysC are inevitably accompanied by an increase in cysteines protease (7), which has direct cytotoxic effects on brain tissue and leads to neuronal death (8). Thus, it is plausible to speculate that CysC's involvement in the clinical prognosis of stroke patients does not simply depend on renal function. However, evidence with a large sample size on this issue is limited (4).

In this analysis of The Third China National Stroke Registry (CNSR-III), we aimed to assess whether CysC was a potential biomarker in the prediction of clinical outcomes among acute ischemic stroke (AIS) patients independent of renal function and to explore the effect of CysC on stroke clinical prognosis in patients with “preserved renal function” [eGFRcreatinine (eGFRcr) ≥60 ml/min/1.73 m^2^].

## Methods

### Study Design and Subjects

**Figure 1:**
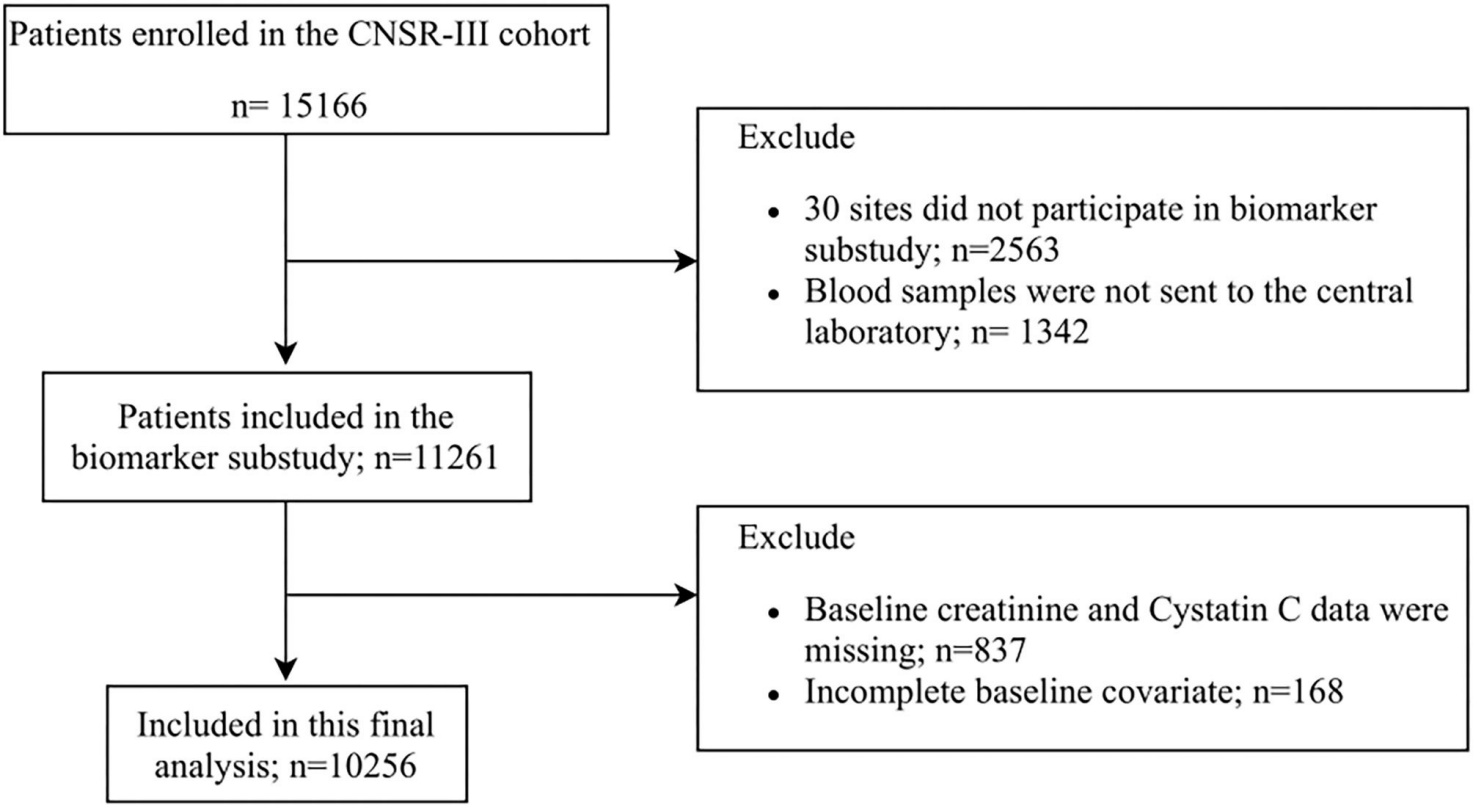
Flowchart of the study.

This study was conducted based on CNSR-III, a nationwide, multicenter, prospective registry study launched in China between August 2015 and March 2018, aiming to evaluate the etiology, imaging, and biological markers of AIS. Detailed descriptions of the CNSR-III study have been reported previously (9). Blood samples were collected from 171 study sites for this prespecified biomarker subgroup analysis. Finally, 10,256 subjects were included in our main analytic sample (Figure 1). The protocol of the CNSR-III study was approved by the ethics committee of Beijing Tiantan Hospital.

Kidney function was estimated by GFR, which was calculated using the latest Chronic Kidney Disease Epidemiology Collaboration (CKD-EPI) equations by creatinine (1). “Renal dysfunction” was defined as eGFR <60 ml/min/1.73 m^2^ based on the National Kidney Foundation Kidney Disease Outcomes Quality Initiative working group definition of kidney disease (10). “Preserved renal function” was defined as eGFRcr ≥60 ml/min/1.73 m^2^. The degree of stenosis was assessed according to computed tomographic angiography (CTA), magnetic resonance angiography (MRA) imaging, or conventional ultrasonography. More than 50% caliber reduction of the intracranial and extracranial artery was defined as intracranial artery stenosis (ICAS) and extracranial artery stenosis (ECAS), respectively. Stroke subtypes are classified according to the modified Trial of Org 10,172 in acute stroke treatment (TOAST). To ensure that the diagnosis standard was consistent, all images were independently evaluated by two trained neuroradiologists blinded to clinical information. A third neuroradiologist was involved for additional assessment if there was disagreement in certain cases.

### Data Collection and Serum Biomarker Measurement

The blood samples were collected on the 1st day of enrollment and transported through the cold chain to the central laboratory in Beijing Tiantan Hospital, where all serum specimens were stored at −80°C until testing was performed. Blood samples were tested uniformly in the central laboratory of Beijing Tiantan Hospital according to the standardized methods. All measurements were performed by laboratory personnel blinded to the study status.

The value of CysC was measured by the immunoturbidimetric method (Roche Cobas c501 analyzer with cystatin C assay); coefficient of variation (CV) of CysC was 2%. Concentrations of serum creatinine were measured by the enzymatic method (sarcosine oxidase-PAP) using a commercial kit (Beckman Coulter, Brea, CA, USA) according to the manufacturer's protocol. The Beckman assay was calibrated to the Roche/Hitachi P module Creatinine Plus enzymatic assay (Roche Diagnostics, Basel, Switzerland), which has an approximate CV of 2%. Low-density lipoprotein cholesterol (LDL-C), high-density lipoprotein cholesterol (HDL-C), and triglyceride (TG) were measured by the enzymatic method. Total cholesterol (TC) testing was the cholesterol oxidase method. Levels of high-sensitivity C-reactive protein (hs-CRP) were measured on Cobas c501 analyzer using the cardiac CRP (latex) high-sensitive assay (Roche, Basel, Switzerland).

### Outcome Assessment

The outcomes were obtained through clinic or telephone in 1-year follow-up. Assessment of endpoints was completed by trained research coordinators who were blinded to patients' baseline clinical information. Patients were contacted over the telephone by trained research coordinators after 1 year. The primary outcome was a combination of all-cause mortality and major disability [modified Rankin scale (mRS) score, 3–6]. Secondary outcomes included stroke recurrence and combined vascular events (including recurrent stroke, myocardial infarction, and vascular death). Stroke recurrence was defined as new onset of focal neurological deficit induced by cerebral ischemic or hemorrhagic events and confirmed by computed tomography/magnetic resonance imaging.

### Statistical Analysis

Baseline characteristics were compared among quartiles of CysC (<0.83 mg/L, 0.83–0.95 mg/L, 0.95–1.10 mg/L, >1.10 mg/L) using the chi-square test for categorical variables and ANOVA or the Kruskal–Wallis test for continuous variables. Logistic regression models and Cox proportional hazards models were performed for stroke outcomes. Variables were adjusted in the multivariable analyses if established as traditional predictors for stroke or associated with CysC in univariate analysis with a value of *p* < 0.05. The backward selection method was adopted in multiple adjustments (Supplementary Table 3). Model 1 adjusted for age, gender, National Institutes of Health Stroke Scale (NIHSS) at admission, antihypertensive agents, hypoglycemic drugs, anticoagulants, ischemic stroke, coronary artery disease, smoking, atrial fibrillation, hs-CRP, TG, TC, and non-HDL-C. Model 2 further adjusted for TOAST subtypes. Model 3 further adjusted for eGFRcr. The association between CysC and stroke patients' clinical outcomes was evaluated using a regression model with restricted cubic splines. The Sankey diagram was used to visualize the mRS score distributions in different CysC quartiles. We performed a sensitivity analysis to explore the differences in the primary outcome between the patients' proportion in different subgroups. Besides, C statistics, integrated discrimination improvement, and net reclassification index were used to assess improvement in model performance by adding CysC to a conventional model (risk factors in model 3) to assess the incremental value of CysC in risk prediction for the prognosis.

All data were analyzed with the SAS version 9.4 software (SAS Institute Inc., Cary, NC). The level of significance was defined as *p* < 0.05 (two-sided).

## Results

### Baseline Characteristics

Of 15,166 stroke patients enrolled in the CNSR-III, 10,256 were included in this analysis. The baseline characteristics of the biomarker cohort vs. those excluded from the overall study population were shown in Supplementary Table 1. Patients included in the analysis were more likely to have lower NIHSS score, a lower rate of statin and hypoglycemic drug uses as compared with excluded patients. Other factors did not differ significantly between the two groups. Among the included participants, 9,508 were “preserved renal function” patients at baseline.

The mean age of the study subjects was 63.0 years; 3,312 (31.7%) patients were female. The median CysC was 0.95 mg/l (interquartile range, 0.83–1.10 mg/l). Subject characteristics grouped by quartiles of serum CysC are listed in Table 1. The participants with higher serum CysC tended to be older, male; had higher prevalence of ischemic stroke, CVD, and atrial fibrillation; and had higher hs-CRP levels than those with lower serum CysC (Table 1).

**Table 1:** Characteristics of all enrolled patients according to CysC quartiles.

Characteristics^*^	**Baseline CysC, mg/l**				***p*-value**
	**Q1 (<0.83) *N* = 2,552 (24.9)**	**Q2 (0.83–0.95) *N* = 2,559 (25.0)**	**Q3 (0.95–1.10) *N* = 2,575 (25.1)**	**Q4 (>1.10) *N* = 2,570 (25.1)**	
No. of patients	2,377 (25.00)	2,377 (25.00)	2,356 (24.78)	2,398 (25.22)	
Age, years, mean ± SD	56.7 ± 10.4	60.9 ± 10.3	63.6 ± 10.5	68.1 ± 10.9	<0.001
Male sex	1,528 (59.9)	1,742 (68.1)	1,856 (72.1)	1,875 (73.0)	<0.001
NIHSS at admission	3.0 (1.0–6.0)	3.0 (1.0–6.0)	3.0 (1.0–6.0)	3.0 (1.0–6.0)	<0.001
0–3	1,331 (52.2)	1,418 (55.4)	1,385 (53.8)	1,337 (52.0)	0.048
≥4	1,221 (47.8)	1,141 (44.6)	1,189 (46.2)	1,233 (48.0)	
BMI	24.6 (22.9–26.6)	24.5 (22.6–26.6)	24.4 (22.5–26.6)	24.5 (22.5–26.6)	0.195
**Medical history**
Ischemic stroke	419 (16.4)	468 (18.3)	617 (24.0)	683 (26.6)	<0.001
Coronary artery disease	189 (7.4)	241 (9.4)	292 (11.3)	393 (15.3)	<0.001
Atrial fibrillation	70 (2.7)	135 (5.3)	180 (7.0)	329 (12.8)	<0.001
Smoking	719 (28.2)	862 (33.7)	886 (34.4)	774 (30.1)	<0.001
Alcohol drinking	329 (12.9)	410 (16.0)	386 (15.0)	326 (12.7)	<0.001
**Laboratory data**
hs-CRP, mg/l	1.4 (0.7–3.7)	1.5 (0.8–3.8)	1.8 (0.8–4.4)	2.7 (1.1–7.7)	<0.001
TG, mmol/l	1.5 (1.1–2.1)	1.3 (1.0–1.8)	1.3 (1.0–1.8)	1.3 (1.0–1.8)	<0.001
TC, mmol/l	4.1 (3.4–4.9)	4.0 (3.3–4.6)	3.9 (3.3–4.7)	3.9 (3.3–4.7)	<0.001
LDL-C, mmol/l	2.3 (1.7–3.0)	2.3 (1.7–3.0)	2.4 (1.8–3.0)	2.3 (1.7–3.0)	0.011
HDL-C, mmol/l	0.9 (0.8–1.1)	0.9 (0.7–1.1)	0.9 (0.8–1.1)	0.9 (0.8–1.1)	0.257
Non-HDL-C, mmol/l	3.2 (2.5–3.9)	3.0 (2.3–3.7)	3.0 (2.4–3.7)	2.9 (2.3–3.7)	<0.001
eGFRcr, ml/min/1.73 m^2^	103.4 (96.8–110.6)	96.5 (90.2–102.5)	90.2 (82.5–97.1)	74.6 (60.7–86.9)	<0.001
**Concomitant medication**
Antihypertensive agents	1,063 (41.7)	1,133 (44.3)	1,180 (45.8)	1,382 (53.8)	<0.001
Statins	2,447 (95.9)	2,460 (96.1)	2,476 (96.2)	2,463 (95.8)	0.909
Hypoglycemic drugs	810 (31.7)	620 (24.2)	580 (22.5)	622 (24.2)	<0.001
Antiplatelets	2,454 (96.2)	2,476 (96.8)	2,480 (96.3)	2,455 (95.5)	0.142
Anticoagulants	251 (9.8)	231 (9.0)	250 (9.7)	298 (11.6)	0.017
TOAST subtypes, no. (%)					<0.001
LAA	678 (26.6)	597 (23.3)	678 (26.3)	644 (25.1)	
CE	0.86 (3.5)	129 (5.0)	161 (6.3)	274 (10.7)	
SVD	515 (20.2)	597 (23.3)	555 (21.6)	469 (18.3)	
Others	1,271 (49.8)	1,236 (48.3)	1,181 (45.9)	1,183 (46.0)	
ICAS or ECAS, no. (%)					<0.001
With	1,122 (51.1)	1,138 (51.8)	1,108 (49.4)	1,016 (45.4)	
Without	1,076 (49.0)	1,058 (48.2)	1,134 (50.6)	1,221 (54.6)	

*SD, standard deviation; Q, quartile; CysC, cystatin C; NIHSS, National Institutes of Health Stroke Scale; BMI, body mass index; hs-CRP, high sensitivity C-reactive protein; TG, triglycerides; TC, total cholesterol; HDL, high-density lipoprotein; LDL, low-density lipoprotein; eGFRcr, creatinine-calculated glomerular filtration rate; LAA, large artery atherosclerosis; CE, cardioembolism; SVD, small vessel disease; ICAS, intracranial arteries stenosis; ECAS, extracranial arteries stenosis; TOAST, Trial of Org 10,172 in acute stroke treatment*.

* *Variables were presented as median (interquartile range) or counts (percentages) unless otherwise indicated*.

### Clinical Outcomes

**Figure 2:**
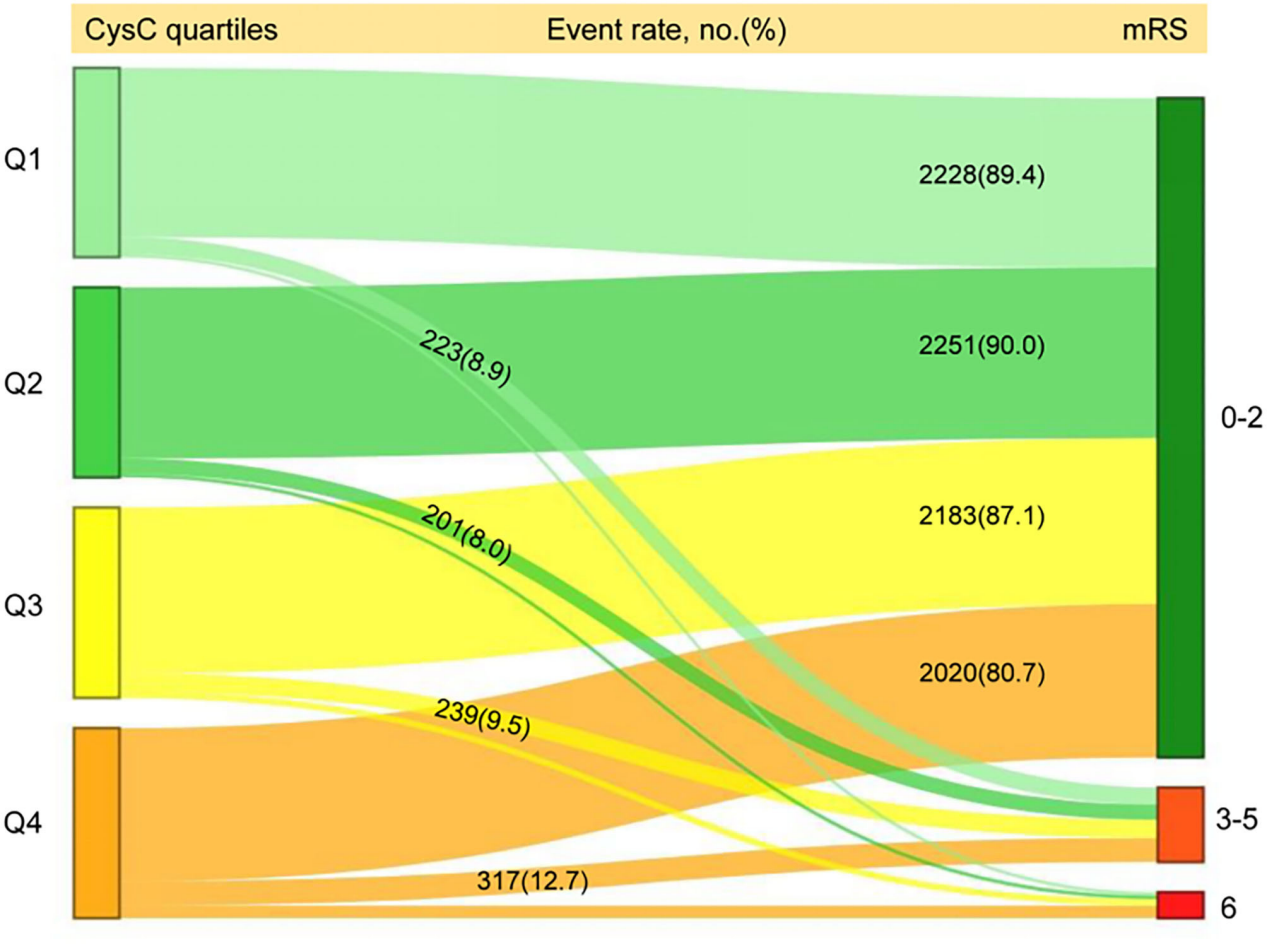
Distribution of 1-year mRS score by CysC quartiles among all the included patients. mRS, modified Rankin Scale; Q, quartile; CysC, cystatin C.

**Figure 3:**
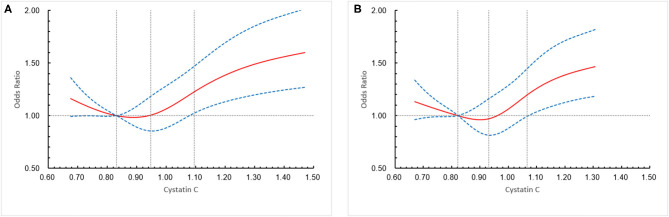
Association of cystatin C (CysC) level with a combination of all-cause mortality and major disability (modified Rankin scale score, 3–6) in all the included patients and “preserved renal function” patients at 1 year. **(A)** All the included patients, **(B) “**preserved renal function” patients. The solid line indicates estimated hazard ratio/odds ratio and the dashed lines the 95% confidence interval bands. Reference is first quartile of CysC. The vertical dashed lines indicate the first, second, and third quartiles of CysC. Data were fitted using a logistic regression model of the restricted cubic spline with 5 knots (the 5th, 25th, 50th, 75th, 95th percentiles) for CysC, adjusting for potential covariates (Model 3). The lowest 5% and highest 5% of participants were not shown in the figures.

A total of 1,321 participants (13.2%) experienced primary outcome (all-cause mortality or major disability) in 1-year follow-up (Table 2). The distribution of 1-year mRS score by CysC quartiles among all the included patients is shown in Figure 2. The cumulative rates of the primary outcome within 1 year among patients with ischemic stroke in the four quartiles of serum CysC (from low to high) were 10.6, 10.0, 12.9, and 19.3%, respectively (Table 2). After adjustment for conventional covariables (model 1) and further adjustment for TOAST subtypes in model 2 and eGFRcr in the full adjusted model (model 3), patients in the first and last CysC quartiles (Q1 and Q4) had worse clinical prognosis (mRS score, 3–6) compared with the second quartile [Q1 vs. Q2: adjusted odds ratio (aOR) 1.29, 95% CI 1.06–1.58, *p* = 0.012; Q3 vs. Q2: aOR 1.12, 95% CI 0.93–1.35, *p* = 0.242; Q4 vs. Q2: aOR 1.35, 95% CI 1.10–1.65, *p* = 0.004]. A U-shaped association was observed between CysC and primary outcome in all the included patients and the “preserved renal function” group (eGFRcr ≥60 ml/min/1.73 m^2^) (Figure 3). Characteristics between the “renal dysfunction” group (eGFRcr <60 ml/min/1.73 m^2^) and the “preserved renal function” group (GFRcr ≥60 ml/min/1.73 m^2^) were shown in Supplementary Table 2.

**Table 2:** Clinical outcomes according to quartiles of serum CysC at 1 year.

**1-year outcomes**	**Event rate, no. (%)^†^**	**Unadjusted model**		**Adjusted model 1^‡^**		**Adjusted model 2^§^**		**Adjusted model 3^||^**	
		HR/OR (95% CI)^*^	***p*-value**	**HR/OR (95% CI)**	***p*-value**	**HR/OR (95% CI)**	***p*-value**	**HR/OR (95% CI)**	***p*-value**
**Primary outcome: All-cause mortality or major disability (modified Rankin scale score, 3–6)**									
Q1 (<0.83)	265 (10.6)	1.07 (0.89–1.29)	0.462	1.29 (1.06–1.57)	0.010	1.27 (1.04–1.55)	0.017	1.29 (1.06–1.58)	0.012
Q2 (0.83–0.95)	250 (10.0)	Reference		Reference		Reference		Reference	
Q3 (0.95–1.10)	323 (12.9)	1.33 (1.12–1.59)	0.001	1.14 (0.95–1.38)	0.156	1.13 (0.94–1.37)	0.190	1.12 (0.93–1.35)	0.242
Q4 (>1.10)	483 (19.3)	2.15 (1.83–2.54)	<0.001	1.42 (1.18–1.70)	<0.001	1.44 (1.18–1.70)	<0.001	1.35 (1.10–1.65)	0.004
**Stroke recurrence**									
Q1 (<0.83)	224 (8.8)	0.94 (0.78–1.13)	0.504	0.96 (0.80–1.16)	0.699	0.95 (0.79–1.14)	0.555	0.96 (0.80–1.16)	0.676
Q2 (0.83–0.95)	239 (9.3)	Reference		Reference		Reference		Reference	
Q3 (0.95–1.10)	265 (10.3)	1.11 (0.93–1.32)	0.239	1.05 (0.88–1.25)	0.628	1.03 (0.87–1.23)	0.719	1.02 (0.85–1.22)	0.827
Q4 (>1.10)	267 (10.4)	1.13 (0.95–1.34)	0.179	0.97 (0.81–1.16)	0.713	0.95 (0.79–1.14)	0.595	0.91 (0.74–1.11)	0.349
**Combined vascular events**									
Q1 (<0.83)	235 (9.21)	0.96 (0.80–1.15)	0.637	0.99 (0.83–1.19)	0.913	0.97 (0.81–1.17)	0.753	0.99 (0.82–1.19)	0.879
Q2 (0.83–0.95)	246 (9.61)	Reference		Reference		Reference		Reference	
Q3 (0.95–1.10)	281 (10.91)	1.15 (0.97–1.36)	0.120	1.07 (0.90–1.27)	0.457	1.06 (0.89–1.26)	0.537	1.04 (0.88–1.24)	0.630
Q4 (>1.10)	290 (11.28)	1.19 (1.00–1.41)	0.045	1.00 (0.84–1.19)	0.980	0.98 (0.82–1.17)	0.851	0.94 (0.77–1.15)	0.540

*CI, confidence interval; Q, quartile; HR, hazard ratio; OR, odds ratio; CysC, cystatin C*.

* *OR for dependence; while HR for stroke recurrence and combined vascular events*.

† *Event rate: no. of patients with event/total no*.

‡ *Model 1 Adjusted for age, gender, NIHSS at admission, antihypertensive agents, hypoglycemic drugs, anticoagulants, ischemic stroke, coronary artery disease, smoking, atrial fibrillation, hs-CRP, TG, TC, Non-HDL-C*.

§ *Model 2 Adjusted for Model 1 + TOAST subtypes*.

|| *Model 3 Adjusted for Model 2 + eGFRcr*.

The cumulative rates of stroke recurrence at 1 year across the four quartiles of serum CysC (from low to high quartile) were 8.8% (n = 224), 9.3% (*n* = 239), 10.3% (*n* = 265), and 10.4% (*n* = 267), respectively, while the combined vascular event rates were 9.21% (*n* = 235), 9.61% (*n* = 246), 10.91% (*n* = 281), 11.28% (*n* = 290) respectively, but there was no statistical difference in the four groups for stroke recurrence and combined vascular events.

### Sensitivity Analysis

Results of sensitivity analysis of the primary outcome are shown in the forest plot in Supplementary Figure 1. There was no heterogeneity in the effects of CysC levels on the primary outcome between subgroups classified by age, gender, TOAST subtypes, and previous stroke history. Of note, the U trend was more pronounced in male, the small artery occlusion group, and subjects without stroke history. Statistical interaction between CysC and gender did not show a significant difference (Supplementary Table 4).

Besides, primary outcomes according to normal ranges of serum CysC (mg/l) (11) were shown in Supplementary Figure 2. Compared with the normal range of CysC, lower and higher range group patients indicated more risk of poor prognosis (Low vs. Normal: aOR 1.60, 95% CI 1.01–2.54, *p* = 0.044; High vs. Normal: aOR 1.10, 95% CI 1.10–1.44, *p* < 0.001). But there was no statistically significant difference among the age subgroups (age <50 or ≥50).

### Incremental Predictive Value of Cystatin C for Prognosis

We evaluated whether CysC would further increase the predictive performance of the models with conventional risk factors on the prognosis of ischemic stroke (Table 3). For all-cause mortality or major disability (mRS score, 3–6) as the outcome of interest, the C statistic by the conventional model improved by the addition of CysC quartile (from 0.765 to 0.791, *p* = 0.014). The risk reclassification appeared to be substantially significant (integrated discrimination improvement 0.13%, *p* = 0.016; quartiles net reclassification index was 13.10%, *p* < 0.001).

**Table 3:** Reclassification and discrimination statistics for outcomes by CysC within 1 year.

**1-year outcomes, no. (%)**		**C statistic**		**IDI, %**		**NRI, ^†^%**	
		**Estimate (95% CI)**	***p*-value**	**Estimate (95% CI)**	***p*-value**	**Estimate (95% CI)**	***p*-value**
All-cause mortality or major disability (modified Rankin scale score, 3–6)	Conventional model^*^	0.776 (0.763–0.790)		–		–	
	Conventional model + CysC quartile	0.778 (0.765–0.791)	0.014	0.13 (0.02–0.23)	0.016	13.10 (7.33–18.86)	<0.001
Stroke recurrence	Conventional model^*^	0.622 (0.603–0.640)		–		–	
							
	Conventional model + CysC quartile	0.623 (0.604–0.641)	0.344	0.02 (−0.01 to 0.05)	0.179	1.45 (−5.09 to 7.99)	0.663
Combined vascular events	Conventional model^*^	0.628 (0.610–0.646)		–		–	
	Conventional model + CysC quartile	0.629 (0.611–0.646)	0.553	0.02 (−0.01 to 0.05)	0.233	1.09 (−5.28 to 7.46)	0.738

*IDI, integrated discrimination improvement; NRI, net reclassification index; BMI, body mass index; LDL, low-density lipoprotein; HDL, high-density lipoprotein; NIHSS, National Institutes of Health Stroke Scale; CysC, cystatin C; hs-CRP, high sensitivity C-reactive protein; TG, triglycerides; TC, total cholesterol; HDL, high-density lipoprotein; LDL, low-density lipoprotein; eGFRcr, creatinine-calculated glomerular filtration rate; TOAST, Trial of Org 10 172 in acute stroke treatment*.

* *Conventional model: age, sex, body mass index, medical history of ischemic stroke, coronary artery disease, atrial fibrillation, smoking and alcohol drinking, NIHSS at admission, laboratory data of hs-CRP, LDL, HDL, TG, TC level, eGFRcr, ml/min/1.73 m^2^, and TOAST subtype*.

† *Patients were divided into four risk categories by CysC quartiles*.

## Discussion

There are several key findings in this study. First, we investigated the association between CysC levels and the prognosis of AIS at 1 year. We demonstrated a U-shaped correlation between CysC and clinical outcome (mortality or major disability) independent of eGFRcr. Second, adding CysC to conventional risk factors (including eGFRcr) could improve risk prediction for clinical outcomes. Another important observation from our study is the fact that half of the subjects in the subset with eGFRcr ≥60 ml/min/1.73 m^2^ still demonstrate the same impact of CysC levels on stroke prognosis. Fourth, the U trend between CysC and the clinical prognosis was more pronounced in the small artery occlusion group and subjects without stroke history.

It was widely acknowledged that CysC is a prominent predictor of CVDs independent of eGFRcr (12–15). To date, several studies have shown the associations of CysC with prognosis and the recurrent vascular event in stroke patients (4, 5, 16, 17). In the previous case-control study, Ni et al. (5) showed that higher plasma CysC levels were independently associated with both ischemic and hemorrhagic stroke and death in 5 years' follow-up. Besides, CysC level was also a useful predictor for early neurological deterioration in AIS patients (17) and short-term outcomes for AIS patients after intravenous tissue plasminogen activator (IV-tPA) therapy (16). On the other hand, previous studies have shown that CysC may provide neuroprotective activities in stroke and neurodegenerative disorders (18, 19). Increasing pieces of evidence revealed that CysC was not only a simple candidate marker of impaired kidney function (20) but also closely associated with congestive heart failure (21, 22), inflammation (23), oxidative stress (19), carotid atherosclerosis (24), and peripheral vascular disease (25) superior to serum creatinine (26). In the current study, we further added evidence of bilateral effects for CysC levels on 1-year prognosis compared to previous studies. Seliger et al. (27) have suggested a quadratic U-shaped association between renal function and subclinical brain infarcts (SBIs) due to small-vessel arteries rather than large-vessel atherosclerosis. We also have discovered that the U-trend was more pronounced in the small artery occlusion group, suggesting the possible effect of small vessel injury on prognosis.

The U-shaped correlation between CysC levels and the clinical outcome means low concentrations of CysC is also detrimental to stroke patients. Additional underlying mechanisms for the seemingly paradoxical outcomes are suggested. CysC is a potent competitive inhibitor of cysteine proteases (28). The balance between cysteine protease and protease inhibitor (CysC) plays an important role in the pathogenesis of cerebral injury and functional rehabilitation (20, 29). Cysteine proteases released after traumatic injury would lead to neuronal death (8). Cathepsin B is a major lysosomal cysteine protease that plays an important role in aging, oxidative stress, inflammation, and apoptosis processes (18, 30). Imbalances between cathepsin B and CysC were involved in atherosclerosis, glomerulosclerosis, and cardiomyopathy with senescence-associated phenotypes (31). It is possible to hypothesize that low levels of CysC are accompanied by an increase in cathepsin content (32), which appears to reflect cell necrosis and brain tissue damage, leading to adverse clinical outcomes, as confirmed in this current real-world clinical cohort analysis.

Furthermore, we proved that serum CysC could significantly improve the predictive power for the primary outcome beyond established traditional risk factors (including eGFRcr), indicating that incremental improvement in risk prediction with CysC is due in part to its non-GFR determinants among ischemic stroke patients. However, there is no significant correlation between CysC levels and 1-year stroke recurrence and combined vascular events in our research.

Besides, patients with elevated CysC seemed more likely to be male, as Q4 has both more numbers and proportion of males than Q2, but there were no significant interactions. Of note, elevated CysC levels (Q4) were more likely to experience poor clinical outcome than Q2 in age ≥65 subgroup in the sensitivity analysis. A cohort from the China Health and Retirement Longitudinal Study also showed that the association between CysC levels and the incidence of ischemic stroke was more pronounced in males or the aged than in females or the young (33). The underlying mechanism needs to be confirmed by further research.

Several studies have investigated the relationship between CysC and the risk of stroke outcomes previously (5, 34, 35). However, evidence from large-scale studies on the relationship between CysC and stroke clinical prognosis is still insufficient. Compared with previous studies, we further added the evidence of a bilateral effect of CysC levels on clinical outcomes after AIS independent of eGFRcr. Our findings corroborated prior studies that suggested that CysC may improve overall risk prediction due in part to its non-GFR determinants. Nonetheless, there are some limitations that need to be interpreted. First, only baseline CysC was analyzed in our study, so we were unable to examine the association of CysC changes with prognosis; further studies with repeated measurement intervals are needed. Second, 4,910 patients of the CNSR-III trial were excluded, and a selection bias may unavoidably be present. However, the baseline characteristics of participants in this study were balanced, suggesting that the selection bias may be minimal. Third, our study has not relied on direct GFR measurement to exclude the compounded effect of GFR on the predictive role of CysC. Fourth, data at 3 months' follow-up were not available; we were unable to determine the relationship between CysC and short-term outcomes. Even when we have tried to adjust for possible confounders such as medication, there are still many factors influencing the long-term prognosis. Finally, only Chinese patients were enrolled in the trial. This limits the generalizability of the findings to a Western population with a different disease pattern or stroke subtypes. Further work is needed to validate our research and seek out possible mechanisms.

## Conclusions

This sub-study of the CNSR-III trial suggests that CysC levels have a bilateral effect on 1-year clinical outcome independent of eGFRcr after ischemic stroke onset. Further prospective studies are needed to validate our findings and to elucidate the potential biological mechanisms.

## Data Availability Statement

The raw data supporting the conclusions of this article will be made available by the authors, without undue reservation.

## Ethics Statement

The studies involving human participants were reviewed and approved by the ethics committee at Beijing Tiantan Hospital (IRB approval number: KY2015-001-01) and all participating centers. The patients/participants provided their written informed consent to participate in this study. Written informed consent was obtained from the individual(s) for the publication of any potentially identifiable images or data included in this article.

## Author Contributions

YW had full access to all the data in the study and takes responsibility for the integrity of the data and the accuracy of the data analysis, designed, and conceptualized this study. YD performed the experiments and drafted the manuscript. LL interpreted the data. ZC revised the manuscript for intellectual content. HL interpreted the data and revised the manuscript. YP conducted the statistical analysis and interpreted the data. JW revised the manuscript for intellectual content. XM, JL, JJ, and XXie performed the experiments and interpreted the data. XXia conducted the statistical analysis. All authors contributed to the article and approved the submitted version.

## Conflict of Interest

The authors declare that the research was conducted in the absence of any commercial or financial relationships that could be construed as a potential conflict of interest.

## Publisher's Note

All claims expressed in this article are solely those of the authors and do not necessarily represent those of their affiliated organizations, or those of the publisher, the editors and the reviewers. Any product that may be evaluated in this article, or claim that may be made by its manufacturer, is not guaranteed or endorsed by the publisher.
