# Supplementary material for: Serum Cystatin C Predicts Stroke Clinical Outcomes at 1 Year Independent of Renal Function
Source: Front Neurol. 2021 Aug 11;12:676872. doi: 10.3389/fneur.2021.676872 (PMC8385197; doi:10.3389/fneur.2021.676872)
Supplement: Supplementary file 1 [file Data_Sheet_1.PDF]

Supplemental Material

Serum Cystatin C Predicts Stroke Clinical Outcomes at 1-year Independent of Renal Function

CONTENTS

Supplementary Table 1. Baseline characteristics between participants with and without blood samples

Supplementary Table 2. Characteristics between 'renal dysfunction' group (eGFRcr<60ml/min/1.73m^2) and 'preserved renal function' group (GFRcr⩾60ml/min/1.73m^2)

Supplementary Table 3. Multivariate analysis of factors influencing CysC elevation(quartiles)

Supplemental table 4. Primary outcome (modified Rankin scale, 3-6) according to serum CysC(mg/l) levels in male and female subgroups at 1-year

Supplementary Figure 1. Primary outcome (modified Rankin scale, 3-6) according to quartiles of serum CysC in subgroups at 1-year

Supplemental Figure 2. Primary Outcomes According to Normal Ranges of Serum CysC(mg/l) and age at 1-year

Supplementary Table 1. Characteristics of participants with and without blood samples

Characteristics*	Patients without laboratory data (N=4910)	Patients with blood samples (N=10256)	P value
Age, y, mean±SD	62.1±11.3	62.3±11.3	0.203
Male gender	3363(68.5)	7001(68.3)	0.775
NIHSS at admission	3(1-6)	3(1-6)	0.001
0-3	2788(33.8)	5472(66.3)	<0.001
≥4	2122(30.7)	4784(69.3)	
BMI	24.5(22.7-26.5)	24.5(22.6-26.6)	0.867
Medical history			
Ischemic stroke	962(19.6)	2187(21.3)	0.014
Coronary artery disease	493(10.0)	1115(10.9)	0.120
Atrial fibrillation	305(6.2)	714(7.0)	0.084
Smoking	1511(30.8)	3241(31.6)	0.304
Alcohol drinking	675(13.8)	1451(14.2)	0.506
Concomitant medication			
Anti-hypertensive agents	2242(45.7)	4758(46.4)	0.399
Statins	4660(94.9)	9846(96.0)	0.002
Hypoglycemic drugs	1160(23.6)	2632(25.7)	0.007
Antiplatelets	4748(96.7)	9865(96.2)	0.115
Anticoagulants	516(10.5)	1030(10.0)	0.375
1-year Outcomes			
Stroke recurrence	478(9.7)	995(9.7)	0.948
Combined vascular events	505(10.29)	1052(10.26)	0.958

Abbreviations：SD, standard deviation; NIHSS, National Institutes of Health Stroke Scale; BMI, Body mass index; mRS, modified Rankin Scale.

* Variables were presented as median (interquartile range) or counts (percentages) unless otherwise indicated.

Supplementary Table 2. Characteristics between 'renal dysfunction' group (eGFRcr<60ml/min/1.73m^2) and 'preserved renal function' group (GFRcr≥60ml/min/1.73m^2)

Characteristics*	CysC at baseline		
	Renal dysfunction group (N=748)	Preserved renal function group (N=9508)	P value †
Age, y, mean±SD	69.0±11.2	61.8±11.2	<0.001
Male gender	455(60.8)	6546(68.8)	<0.001
BMI	24.2(22.3-26.3)	24.5(22.6-26.6)	0.390
NIHSS at admission	4(2-7)	3(1-6)	<0.001
Medical history			
Ischemic stroke	218(29.1)	1969(20.7)	<0.001
Coronary artery disease	123(16.4)	992(10.4)	<0.001
Atrial fibrillation	430(57.5)	4328(45.5)	<0.001
Smoking	150(20.1)	309(32.5)	<0.001
Alcohol drinking	60(8.0)	1391(14.6)	<0.001
Laboratory data			
hs-CRP, mg/l	3.5(1.2-11.2)	1.7(0.8-4.4)	<0.001
TG, mmol/l	1.6(1.1-2. 2)	1.4(1.0-1.8)	<0.001
TC, mmol/l	4.3(3.5-5.6)	4.0(3.3-4.7)	<0.001
LDL-C, mmol/l	2.5(1.9-3.7)	2.3(1.7-3.0)	<0.001
HDL-C, mmol/l	1.0(0.8-1.2)	0.9(0.8-1.1)	<0.001
eGFRcr, ml/min/1.73 m2	49.6(40.0-55.8)	94.3(84.9-102.5)	<0.001
CysC, mg/l	1.4(1.2-1.8)	0.9(0.8-1.1)	<0.001
Concomitant medication			
Anti-hypertensive agents	430(57.5)	4328(45.5)	<0.001
Statins	713(95.3)	9133(96.1)	0.323
Hypoglycemic drugs	217(29.0)	2415(25.4)	0.030
Antiplatelets	709(94.8)	9156(96.3)	0.037
Anticoagulants	111(14.8)	919(9.7)	<0.001

Abbreviations：SD, standard deviation; CysC, Cystatin C; NIHSS, National Institutes of Health Stroke Scale; BMI, Body mass index; hs-CRP denotes high sensitivity C reactive protein; TG triglycerides; TC, total cholesterol; HDL, high-density lipoprotein; LDL, low-density lipoprotein; eGFRcr, creatinine-calculated glomerular filtration rate.

* Variables were presented as median (interquartile range) or counts (percentages) unless otherwise indicated.

† P-value for difference between $eGFR_{MDRD}$≥60ml/min/1.73m^2 and $eGFR_{MDRD}$<60ml/min/1.73m^2.

Supplementary Table 3. Multivariate analysis of factors influencing CysC elevation(quartiles)

	CysC elevation(quartiles)	
Characteristics*	Odds ratio(95%CI)	P value
Age (in 10 y)	1.09(1.04-1.14)	<0.001
Male gender	0.60(0.54-0.65)	<0.001
NIHSS at admission	0.98(0.97-0.99)	0.006
Medical history		
Ischemic stroke	1.27(1.16-1.40)	<0.001
Coronary artery disease	1.17(1.04-1.33)	<0.001
Atrial fibrillation	1.37(1.16-1.62)	<0.001
Smoking	1.49(1.36-1.63)	0.003
Concomitant medication		
Anti-hypertensive agents	1.28(1.19-1.38)	0.023
Hypoglycemic drugs	0.82(0.75-0.90)	<0.001
Anti-coagulants	0.87(0.76-0.99)	<0.001
Laboratory data		
hs-CRP (per 10 units), mg/l	1.03(1.01-1.04)	0.004
TG, mmol/l	0.75(0.71-0.79)	<0.001
TC, mmol/l	0.49(0.43-0.57)	<0.001
Non-HDL-C, mmol/l	1.89(1.62-2.20)	<0.001
eGFRcr (per 10 units), ml/min/1.73 m^2	0.39(0.37-0.40)	<0.001

Abbreviations：CysC, Cystatin C; NIHSS, National Institutes of Health Stroke Scale; LAA, Large artery atherosclerosis; CE, Cardio embolism; SVD, Small vessel disease; hs-CRP denotes high sensitivity C reactive protein; TG, triglycerides; TC, total cholesterol; HDL, high-density lipoprotein; eGFRcr, creatinine-calculated glomerular filtration rate.

*Variables were presented as median (interquartile range) or counts (percentages) unless otherwise indicated. For the analysis of the associations with serum CysC levels, all variables showing a univariate association with serum CysC (Table 1) were included in the original backward multivariate model.

Supplemental Table 4. Primary outcome (modified Rankin scale, 3-6) according to serum CysC(mg/l) levels in male and female subgroups at 1-year

Subgroup	Baseline CysC,mg/l	No. of patients with event/total no.	Adjusted OR (95%CI)	P value	P interaction
Male	Q1(<0.83)	149(10.2)	1.39(1.07-1.80)	0.013	0.752
	Q2(0.83-0.95)	149(8.5)	reference		
	Q3(0.95-1.10)	214(11.9)	1.21(0.95-1.53)	0.120	
	Q4(>1.10)	315(17.4)	1.36(1.05-1.75)	0.019	
Female	Q1(<0.83)	114(11.6)	1.30(0.95-1.77)	0.098	
	Q2(0.83-0.95)	108(12.9)	reference		
	Q3(0.95-1.10)	112(16.2)	1.09(0.80-1.49)	0.570	
	Q4(>1.10)	160(24.2)	1.48(1.06-2.08)	0.022	

Adjusted for age, gender, NIHSS at admission, anti-hypertensive agents, hypoglycemic drugs, anticoagulants, ischemic stroke, coronary artery disease, atrial fibrillation, smoking, TOAST subtypes, hs-CRP, TG, TC, Non-HDL-C, eGFRcr

Supplementary Figure 1. Primary outcome (modified Rankin scale, 3-6) according to quartiles of serum CysC(mg/l) in subgroups at 1-year

1–Year Primary Outcome (modified Rankin scale, 3–6)	Event rate, no. (%) *	Adjusted OR (95% CI)†	P value
**Age≥65 yr**			
Q1(<0.83 mg/l)	97(17.5)	1.28(0.95–1.72)	0.112
Q2(0.83–0.95 mg/l)	140(15.2)	reference	reference
Q3(0.95–1.10 mg/l)	219(18.3)	1.18(0.92–1.51)	0.183
Q4(>1.10 mg/l)	387(24.1)	1.38(1.06–1.78)	0.015
**NIHSS 0–3**			
Q1(<0.83 mg/l)	48(3.7)	1.32(0.87–2.00)	0.200
Q2(0.83–0.95 mg/l)	48(3.5)	reference	reference
Q3(0.95–1.10 mg/l)	92(6.8)	1.66(1.15–2.39)	0.007
Q4(>1.10 mg/l)	136(10.5)	1.77(1.20–2.62)	0.004
**Male**			
Q1(<0.83 mg/l)	151(10.1)	1.35(1.04–1.75)	0.023
Q2(0.83–0.95 mg/l)	145(8.6)	reference	reference
Q3(0.95–1.10 mg/l)	212(11.7)	1.17(0.92–1.48)	0.209
Q4(>1.10 mg/l)	319(17.4)	1.34(1.04–1.74)	0.023
**Large artery atherosclerosis**			
Q1(<0.83 mg/l)	112(17.1)	1.31(0.93–1.84)	0.124
Q2(0.83–0.95 mg/l)	81(14.2)	reference	reference
Q3(0.95–1.10 mg/l)	116(17.6)	1.14(0.82–1.60)	0.436
Q4(>1.10 mg/l)	149(23.7)	1.40(0.97–2.02)	0.076
**Cardioembolism**			
Q1(<0.83 mg/l)	11(13.4)	1.33(0.55–3.19)	0.525
Q2(0.83–0.95 mg/l)	19(15.0)	reference	reference
Q3(0.95–1.10 mg/l)	30(19.4)	1.27(0.65–2.50)	0.488
Q4(>1.10 mg/l)	59(22.1)	0.81(0.40–1.63)	0.558
**Small artery occlusion**			
Q1(<0.83 mg/l)	26(5.1)	2.30(1.17–4.51)	0.016
Q2(0.83–0.95 mg/l)	15(2.6)	reference	reference
Q3(0.95–1.10 mg/l)	30(5.6)	1.82(0.94–3.52)	0.076
Q4(>1.10 mg/l)	52(11.5)	3.31(1.68–6.50)	0.001
**Without Stroke history**			
Q1(<0.83 mg/l)	190(9.2)	1.31(1.04–1.66)	0.024
Q2(0.83–0.95 mg/l)	171(8.5)	reference	reference
Q3(0.95–1.10 mg/l)	203(10.8)	1.11(0.88–1.39)	0.387
Q4(>1.10 mg/l)	316(17.6)	1.41(1.11–1.80)	0.005

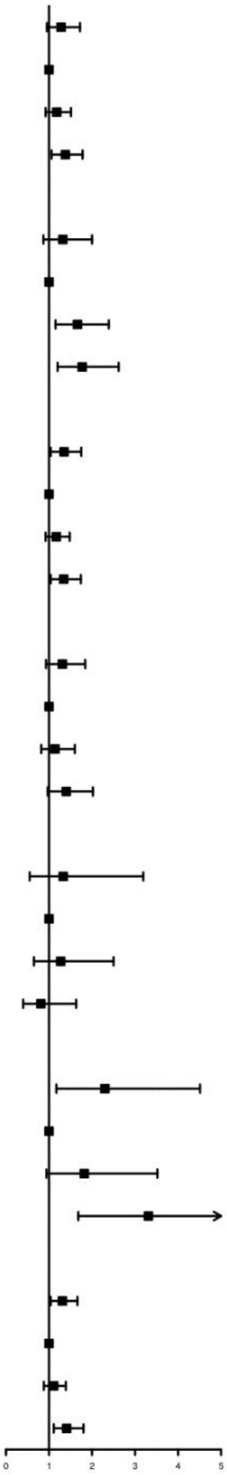

Abbreviations: CI, confidence interval; Q, quartile; and OR, odds ratio; yr, years; NIHSS, National Institutes of Health Stroke Scale.

* Event rate：no. of patients with event/total no.

†Adjusted for age, gender, NIHSS at admission, anti-hypertensive agents, hypoglycemic drugs, anticoagulants, ischemic stroke, coronary artery disease, atrial fibrillation, smoking, TOAST subtypes, hs-CRP, TG, TC, Non-HDL-C+ eGFRcr.

Supplemental Figure 2. Primary Outcomes According to Normal Ranges of Serum CysC(mg/l) and age at 1-year.

Primary outcome: All–cause mortality or major disability (modified Rankin scale, 3– 6)	Event rate, no. (%) *	Adjusted OR (95% CI)†	P value
**All the patients**			
Low(<0.60 mg/l)	25(12.4)	1.60(1.01–2.54)	0.044
Normal(0.60–1.00 mg/l)	612(10.4)	reference	reference
High(>1.00 mg/l)	684(17.4)	1.26(1.10–1.44)	<0.001
**Age<50 years**			
Low(<0.53 mg/l)	2(7.1)	2.77(0.58–13.16)	0.200
Normal(0.53–0.92 mg/l)	56(6.6)	reference	reference
High(>0.92 mg/l)	26(6.1)	1.02(0.61–1.70)	0.956
**Age≥50 years**			
Low(<0.58 mg/l)	11(10.8)	1.05(0.54–2.04)	0.895
Normal(0.58–1.02 mg/l)	605(11.5)	reference	reference
High(>1.02 mg/l)	621(18.5)	1.21(1.05–1.39)	0.007

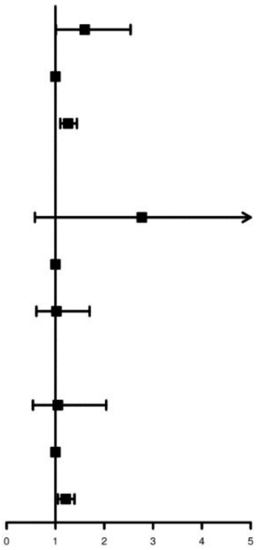

Abbreviations: CI, confidence interval; and OR, odds ratio

* Event rate：no. of patients with event/total no.

†Adjusted for age, gender, NIHSS at admission, anti-hypertensive agents, hypoglycemic drugs, anticoagulants, ischemic stroke, coronary artery disease, atrial fibrillation, smoking, hs-CRP, TG, TC, Non-HDL-C
